# Association of Neonatal Hypothermia with Morbidity and Mortality in a Tertiary Hospital in Malawi

**DOI:** 10.1093/tropej/fmz086

**Published:** 2020-03-16

**Authors:** Frank Phoya, Josephine Langton, Queen Dube, Pui-Ying Iroh Tam

**Affiliations:** 1 Department of Paediatrics, Queen Elizabeth Central Hospital, Blantyre, Malawi; 2 Department of Paediatrics, University of Malawi College of Medicine, Blantyre, Malawi; 3 Paediatrics and Child Health Research Group, Malawi-Liverpool Wellcome Trust Clinical Research Programme, Blantyre, Malawi; 4 Department of Clinical Sciences, Liverpool School of Tropical Medicine, Liverpool, UK

**Keywords:** neonatal hypothermia, risk factors, Kangaroo mother care, neonatal morbidity, hospital mortality

## Abstract

**Objectives:**

To evaluate associations with neonatal hypothermia in a tertiary-level neonatal unit (NU) in Malawi.

**Methods:**

Neonates with a birth weight >1000 g were recruited and temperatures were recorded 5 min after birth, on admission and 4 h thereafter. Clinical course and outcome were reviewed. Data were analysed using Stata v.15 and *p* < 0.05 was considered statistically significant.

**Results:**

Between August 2018 to March 2019, 120 neonates were enrolled, and 112 were included in the data analysis. Hypothermia at 5 min after birth was noted in 74%, 77% on admission to the NU and 38% at 24 h. Neonates who had hypothermia 5 min after birth were more likely to have hypothermia on admission to the NU compared with normothermic subjects (*p* < 0.01). All neonates with hypothermia on admission to the NU died (100 vs.72%, *p* = 0.02), but hypothermia at 5 min nor at 24 h were not associated with mortality. After adjusting for potential confounders, the odds ratio of hypothermia at 5 min for hypothermia on admission to NU was 13.31 (95% CI 4.17–42.54).

**Discussion:**

A large proportion of hospitalized neonates is hypothermic on admission and has associated morbidity and mortality. Our findings suggest that a strong predictor of mortality is neonatal hypothermia on admission to the NU, and that early intervention in the immediate period after delivery could decrease the incidence of hypothermia and reduce associated morbidity and mortality.

## BACKGROUND

In keeping with the global trend, Malawi achieved the Millennium Development Goal 4, reducing under five mortality between 1990 and 2015 by two-thirds. However, the neonatal mortality has remained high, and is currently at 27 per 1000 live births. In response to this slow reduction in neonatal mortality rate, Malawi adopted the ‘Newborn Action Plan’ from Every Newborn Action Plan, which was launched at the World Health Assembly in June 2014. This plan puts in place strategies to accelerate the reduction of preventable newborn deaths and stillbirths in Malawi, with the ultimate goal of ending all preventable newborn deaths, including stillbirths.

One of the major risk factors for morbidity and mortality in the first 28 days of life is neonatal hypothermia. Hypothermia has also been shown to be a risk factor for neonatal sepsis, intra-ventricular haemorrhage, and necrotizing enterocolitis [1]. In low resource settings, a high incidence of hypothermia has been documented in hospitals immediately following birth. Studies conducted in Zambia and Zimbabwe in the late 1990s to 2000s identified hypothermia in 44–51% of sick newborns on admission to the neonatal unit (NU) [2, 3]; however, more recent data on hypothermia in NUs in sub-Saharan Africa are lacking. Furthermore, few studies have evaluated whether hypothermia after birth, on admission to the NU, or later, has the most impact on neonatal outcomes.

Queen Elizabeth Central Hospital (QECH), Blantyre Malawi is a government funded tertiary-level referral hospital, serving the southern region of Malawi. Within the NU care delivered to newborns includes: warmth, feeding, intravenous fluids, parenteral antibiotics, oxygen and continuous positive airway pressure (CPAP). There is no access to more sophisticated interventions such as surfactant therapy and mechanical ventilation. Kangaroo mother care (KMC) is initiated after achieving stabilization, or intermittent KMC is done if the baby is still considered high risk. This study aimed to address this gap by documenting neonatal hypothermia and associated morbidity and mortality in a neonatal care unit in Malawi, and to determine which among hypothermia at 5 min, on admission to the NU, or at 24 h, had the highest association with morbidity and mortality.

## METHODS

This prospective observational study recruited neonates from the labour ward who were subsequently admitted to the NU. All newborns with a gestational age of ≥28 weeks, or birth weight of ≥1000 g, were eligible for inclusion. Newborns with congenital abnormalities were excluded. Written informed consent was obtained from the mother. Ethics approval was awarded by the University of Malawi College of Medicine Research Ethics Committee.

In accordance to standard unit practice all babies had a temperature recorded within 15 min of delivery and on admission to NU. Temperatures were recorded using a digital axillary thermometer. Babies were recruited following admission into the NU. Anonymized data were collated from the standard daily observation charts. For the first 24 h of admission this comprised 4 h vital signs (temperature, heart rate, respiratory rate and oxygen saturation) and 6 h blood glucose checks. For the remainder of the admission it comprised 4 h vital signs, 12 h blood glucose checks and daily weight check, in accordance with standard practice. Patients were reviewed by a clinician twice a day or as determined by their clinical condition. Demographic information, concomitant conditions, clinical course and outcome were also recorded on the anonymized case report form. Daily environmental temperature readings were taken on both the NU and labour ward. The WHO definition for hypothermia (<36.5°C) was adopted for this study.

### Statistical analysis

Descriptive statistics were shown as frequencies and percentages or as a mean (±SD), as appropriate. Primary outcome measures were neonatal temperature at 5 min after birth, on admission to the NU, and at 24 h of life. Secondary outcome measures were associated morbidities, need for respiratory support, hospital length of stay and hospital mortality. Chi-square and Fisher’s exact test were used. Linear and logistic regression analyses were conducted using Stata v.15 (Statacorp, College Station, TX) and odds ratios (OR) were calculated after controlling for potential confounders prematurity, sex and low Apgar scores at 1 min. The *p* values ≤ 0.05 were considered statistically significant.

## RESULTS

Between August 2018 and March 2019, a total of 120 newborns were recruited. Eight newborns were lost to follow-up, meaning 112 completed files were included for analysis. Maternal median age was 25 years, and 13% were HIV positive (Table 1). Of the neonates, 48% were male, 55% were low-birth weight (<2.5 kg) and 16% were very low-birth weight (<1.5 kg). In total 74% of neonates had hypothermia 5 min after birth, 77% on admission to NU, 31% at 6 h, 29% at 12 h and 38% at 24 h. Drying after delivery was documented in 96% of cases, 7% received skin-to-skin contact, 4% breastfed immediately after birth, and less than half received KMC, their own bedding or hat (46, 34 and 42%, respectively).

**Table 1:** Maternal and neonatal characteristics

	*n* = 112
Maternal characteristics	
Maternal age, years (mean, SD)	25.8 (6.8)
Maternal HIV status (%)	
Infected	14 (13)
Unknown	1 (0.9)
Mode of delivery (%)	
Caesarean section	34 (31)
Types of pregnancy (%)	
Singleton	98 (88)
Antenatal visits (%)	
4 complete visits	31 (30)
≤3 antenatal visits	71 (69)
Prematurity (%)	34 (31)
Steroid use in pregnancy (%)	14 (13)
Prolonged rupture of membranes (%)	11 (10)
Infection during pregnancy (%)	5 (4)
Hypertension in pregnancy (%)	13 (12)
Newborn characteristics	
Male (%)	54 (48)
HIV Exposed (%)	14 (13)
Low-birth weight (<2.5 kg, %)	62 (55)
Very low-birth weight (<1.5 kg, %)	18 (16)
Apgar score <6 at 1 min (%)	47 (42)
Apgar score <6 at 5 min (%)	73 (65)
Apgar score <6 at 10 min (%)	6 (7)
Small for gestational age (%)	12 (11)
Intrauterine growth restriction (%)	18 (16)
Inpatient care	
Drying after delivery (%)	108 (96)
Skin-to-skin contact after birth (%)	8 (7)
Breastfeeding after birth (%)	4 (4)
Warm transport to nursery (%)	103 (92)
NICU room temperature, °C, mean (SD)	29.3 (1.4)
Hypothermia (%)	
Five minutes after birth	83 (74)
On admission to NU	86 (77)
6 h after admission	19/62 (31)
12 h after admission	13/45 (29)
24 h after admission	24/63 (38)
Before death/discharge	40/105 (38)
Glucose level on admission, mg/dl, mean (SD)	102.8 (34.5)
Neonatal comorbidity (%)	
RDS	42 (38)
Transient tachypnoea of the newborn	26 (23)
Birth asphyxia	12 (11)
Neonatal sepsis with use of antibiotics	34 (30)
Neonatal jaundice	8 (7)
Meconium aspiration	11 (10)
Received KMC (%)	51 (46)
Provided own bedding (%)	49 (44)
Provided own clothing (%)	38 (34)
Provided own hat (%)	47 (42)
Respiratory support (%)	
Oxygen supplementation	63 (57)
CPAP	18 (16)
Neonatal outcomes	
Length of admission, days, mean (SD)	5.5 (6.3)
Hospital mortality (%)	15 (14)

**Fig. 1.**
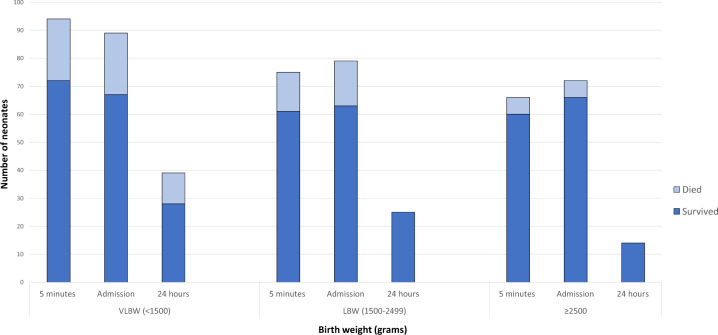
Frequency of hypothermia at 5 min, on admission to NICU, and at 24 h, by birth weight, stratified by those that died vs. survived. LBW, low-birth weight; VLBW, very low-birth weight.

The two most common comorbidities were respiratory distress syndrome (RDS, 38%) and transient tachypnoea of the newborn (23%). Over half of the subjects (57%) required oxygen support during their hospital stay, which was a mean of 5.5 days (±6.3 days). Fifteen neonates died (14%), and this number was higher among lower birth weight neonates with hypothermia (Fig. 1). Among the neonates, 38% of the neonates were hypothermic on their last temperature reading before discharge or death.

Neonates who were hypothermic 5 min after birth were more likely to have hypothermia on admission to the NU compared with those that were normothermic (85 vs. 15%, *p* < 0.01), more likely to have RDS (88 vs. 12%, *p* < 0.01); more likely to receive KMC (84 vs. 16%, *p* = 0.02); more likely to be provided a hat (85 vs. 15%, *p* = 0.02); and more likely to have a longer hospital stay (87 vs. 13%, *p* = 0.03; Table 2). When compared with those that were normothermic on admission, neonates with hypothermia on admission to the NU were more likely to have a diagnosis of transient tachypnoea of the newborn (62 vs. 38%, *p* = 0.04) and to receive oxygen supplementation (86 vs. 14%, *p* = 0.01). Longer hospital stay was documented among neonates with hypothermia on admission to NU (86 vs. 14%), although this was not statistically significant. All the neonates who died had hypothermia on admission (100 vs. 0%, *p* = 0.02).

**Table 2:** Association between neonatal hypothermia at 5 min, on admission to NU and at 24 h, and clinical features and outcomes

	At 5 min (*n* = 112)			On admission (*n* = 111)			At 24 h (*n* = 53)		
	Present (*n* = 83)	Absent (*n* = 29)	*p*-value	Present (*n* = 86)	Absent (*n* = 25)	*p*-value	Present (*n* = 24)	Absent (*n* = 29)	*p*-value
Perinatal characteristics (%)										
Caesarean section	24/34 (71)	10/34 (29)	0.62	24/34 (71)	10/34 (29)	0.34	8/18 (44)	10/18 (56)	0.60
Prematurity	29/34 (85)	5/34 (15)	0.07	28/34 (82)	6/34 (18)	0.36	11/24 (36)	13/24 (54)	0.32
Gestational age <34 weeks	49/62 (79)	13/62 (21)	0.19	49/62 (79)	13 (21)	0.53	15/38 (39)	23/38 (61)	0.78
Low-birth weight <2.5 kg	33/44 (75)	11/44 (44)	0.06	50/62 (81)	12/62 (19)	0.35	17/40 (43)	23/40 (58)	0.63
Very low-birth weight <1.5 kg	17/18 (94)	1/18 (6)	0.06	16/18 (89)	2/18 (11)	0.35	7/16 (44)	9/16 (56)	0.63
Apgar score <6 at 1 min	32/47 (68)	15/47 (32)	0.22	40/47 (85)	7/47 (15)	0.08	8/24 (33)	16/24 (67)	0.54
Apgar score <6 at 5 min	51/73 (70)	22/73 (30)	0.16	59/73 (81)	14/73 (19)	0.17	15/44 (34)	29/44 (66)	0.32
Apgar score <6 at 10 min	5/6 (83)	1/6 (17)	0.51	6/6 (100)	0/6 (0)	0.12	0/2 (0)	2/2 (100)	0.29
Neonatal comorbidity (%)										
Hypothermia on admission to NU	73/86 (85)	13/86 (15)	<0.01	–	–	–	19/52 (37)	33/52 (63)	0.58
Suspected sepsis	21/28 (75)	7/28 (25)	0.90	22/28 (79)	6/28 (21)	0.80	6/23 (26)	17/23 (74)	0.14
RDS	37/42 (88)	5/42 (12)	<0.01	35/42 (83)	7/42 (17)	0.20	15/33 (45)	18/33 (55)	0.21
Birth asphyxia	9/12 (75)	3/12 (25)	0.94	11/12 (92)	1/12 (8)	0.20	0/8 (0)	8/8 (100)	0.02
Transient tachypnoea of the newborn	16/26 (62)	10/26 (39)	0.10	16/26 (62)	10/26 (38)	0.04	4/9 (44)	5/9 (56)	0.67
Inpatient care (%)										
Received O_2_	53/63 (84)	10/63 (16)	<0.01	54/63 (86)	9/63 (14)	0.01	16/43 (37)	27/43 (63)	0.83
Received CPAP	16/18 (89)	2/18 (11)	0.12	17/18 (94)	1/18 (6)	0.05	6/16 (38)	10/16 (63)	0.96
Received KMC	43/51 (84)	8/51 (16)	0.02	42/51 (82)	9/51 (18)	0.20	15/36 (42)	21/36 (58)	0.50
Provided own bedding	39/49 (80)	10/49 (20)	0.24	40/49 (82)	9/49 (18)	0.26	17/37 (46)	20/37 (54)	0.12
Provided own clothing	30/38 (79)	8/38 (21)	0.40	29/38 (76)	9/38 (24)	0.93	15/32 (47)	17/32 (53)	0.15
Provided own hat	40/47 (85)	7/47 (15)	0.02	40/47 (85)	7/47 (15)	0.08	17/35 (49)	18/35 (51)	0.06
Neonatal outcomes (%)										
Duration of stay >5 days	32/37 (86)	5/37 (14)	0.03	32/37 (86)	5/37 (14)	0.08	12/33 (36)	21/33 (64)	0.77
Hospital mortality	13/15 (87)	2/15 (13)	0.20	15/15 (100)	0/15 (0)	0.02	2/7 (29)	5/7 (71)	0.53

At 24 h of life, the only significant association with neonatal hypothermia was birth asphyxia, and none of the babies with birth asphyxia had hypothermia (0 vs. 100%, *p* = 0.02). Neonates that were provided their own hat in the NU were less likely to have hypothermia at 24 h (49 vs. 51%), although this was not statistically significant. Hypothermia at 24 h inversely correlated with longer hospital stay (36 vs. 64%) and hospital mortality (29 vs. 71%), although again neither were statistically significant.

Regarding mortality (Table 3), hospital deaths were significantly associated with low Apgar scores <6 at 1 min (73 vs. 63%, *p* < 0.01) and 10 min (36 vs. 7%, *p* < 0.01), hypothermia on admission to the NU (100 vs. 72%, *p* = 0.02), RDS (60 vs. 32%, *p* = 0.04), birth asphyxia (27 vs. 9%, *p* = 0.04) and receipt of respiratory support (*p* < 0.01). A higher proportion of neonates who died had hypothermia at 5 min (87 vs. 29%); however, this was not statistically significant. Hypothermia at 5 min had an OR of 2.65 (95% CI 0.56–12.59) for death that was not statistically significant (*p* = 0.218).

**Table 3:** Neonatal mortality and association with clinical features and management

Characteristic	Died (*n* = 15)	Survived (*n* = 93)	*p*-value
Perinatal characteristics (%)			
Prematurity	9 (60)	41 (44)	0.25
Low-birth weight <2.5 kg	11 (74)	47 (51)	0.47
Very low-birth weight <1.5kg	4 (27)	12 (13)	0.19
Apgar score < 6 at 1 min	11 (73)	59 (63)	<0.01
Apgar score < 6 at 5 min	12 (80)	34 (37)	0.21
Apgar score <6 at 10 min	5/14 (36)	1/70 (7)	<0.01
Neonatal comorbidity			
Hypothermia at 5 min	13 (87)	27 (29)	0.20
Hypothermia on admission to NU	15 (100)	67 (72)	0.02
Hypothermia at 24 h	2 (29)	22 (41)	0.54
Hypothermia before discharge	4/13 (31)	36/89 (40)	0.50
Suspected sepsis	2 (13)	25 (27)	0.26
RDS	9 (60)	30 (32)	0.04
Birth asphyxia	4 (27)	8 (9)	0.04
Transient tachypnoea of the newborn	0 (0)	26 (28)	0.02
Inpatient care (%)			
Respiratory support			
Oxygen	15 (100)	45 (48)	<0.01
CPAP	7 (47)	9 (10)	<0.01
Received KMC	5 (33)	44 (47)	0.31
Provided own bedding	7 (47)	39 (42)	0.73
Provided own clothing	3 (20)	33 (35)	0.24
Provided own hat	4 (27)	41 (44)	0.20
Outcomes (%)			
Duration of stay >5 days	4 (27)	31 (33)	0.61

After adjusting for low Apgar scores at 1 min, prematurity and sex, hypothermia 5 min after birth had an OR of 13.31 (95% CI 4.17–42.54) for hypothermia on admission to the NU, and hypothermia on admission to the NU had an OR of 0.05 (95% CI 0.02–1.25) for receipt of CPAP. Apgar scores <6 at 1 min had an OR of 5.66 (95% CI 1.55–20.70) for hospital mortality (Table 4), and receipt of CPAP had an OR of 0.05 (95% CI 0.009–0.31) for mortality.

**Table:** Table 4 Multivariable analysis of association between hypothermia, comorbidity and outcomes

Outcome	Unadjusted OR (95% CI)	Adjusted^a^ OR (95% CI)
Hypothermia 5 min after birth		
Low-birth weight	2.15 (1.10–4.19)	2.17 (0.91–5.18)
Hypothermia on NU admission	8.98 (3.35–24.09)	13.31 (4.17–42.54)
RDS	0.26 (0.09–0.74)	0.26 (0.07–0.99)
Receipt of O_2_	0.30 (0.12–0.72)	0.32 (0.13–0.84)
Receipt of KMC	0.35 (0.14–0.89)	0.38 (0.12–1.21)
Hypothermia on NU admission		
Receipt of O_2_	0.31 (0.13–0.79)	0.34 (0.12–0.94)
Inpatient neonatal mortality		
Apgar score <6 at 1 min	4.77 (1.34–16.16)	5.66 (1.55–20.70)
CPAP	0.12 (0.03–0.45)	0.05 (0.009–0.31)

CI, confidence interval; CPAP, continuous positive airway pressure; KMC, kangaroo mother care; NU, neonatal unit; OR, odds ratio.

a Adjusted for Apgar score <6 at 1 min, prematurity, sex.

## DISCUSSION

The incidence of hypothermia 5 min after birth was extremely high and associated with hypothermia on admission to the NU, RDS, receipt of oxygen supplementation, receipt of KMC and of own hat and longer hospital stay. Hypothermia on admission was associated with receipt of oxygen supplementation and death. The only significant association identified with hypothermia at 24 h was birth asphyxia.

These results demonstrate the high incidence of hypothermia in our setting and that this issue affects a larger population beyond those that are preterm and low-birth weight. These findings support previous studies that highlight the importance of thermal care in the newborn in the immediate period after birth. In other settings, hypothermia on admission to NUs was associated with a 1.26- to 1.72-fold greater risk for hospital mortality [4, 5]. In recognition of the importance of neonatal hypothermia as a risk factor for neonatal morbidity and mortality, WHO introduced practical guidelines for clinicians in 2007 for thermal protection. These emphasize the need for drying the baby immediately after birth, skin-to-skin contact with the mother and immediate breastfeeding. In addition, newborns should be wrapped in a warm cloth and given a hat, and provided warm transportation during transfer from delivery room to the NU [1]. A study in Brazil demonstrated that use of plastic bag/wrap independently decreased chances of hypothermia at 5 min by 47% in preterm infants [5], and a Cochrane review found that hypothermia on admission decreased by 34% in preterm infants with gestational age <28 weeks when a plastic bag wrap was used soon after birth [6]. In preterm infants, the use of a cotton cap after birth decreased hypothermia on admission [5].

In our cohort, we documented drying after delivery in 96% of cases, but only 7% received skin-to-skin contact and 4% breastfed immediately after birth. We did not have sufficient numbers to evaluate the association of these interventions with mortality. Despite the low-resource setting of this study (lack of heating in delivery room, environmental temperature optimized only for the mother and staff, poor hypothermia prevention on delivery, poor baby wrapping, lack of heated transportation to the NU), these results highlight an opportunity for simple, low-cost interventions that can impact clinical outcome.

Although evaluating KMC, hypothermia at 5 min after birth was associated with an OR of 0.34 for receipt of KMC, but after adjusting for potential confounders the association was no longer significant. It may have been that hypothermia reflected other comorbidities that required more intensive monitoring, and therefore KMC could not be practiced; another possibility is that staff shortages mean these results reflect a continuum of resource-constrained care. In particular, KMC has been shown to be a viable intervention for treating hypothermia, and more importantly is a low-cost measure in our setting since we have lack of other thermal care resources (warmed incubator transportation, plastic wrap, enough radiant warmers). A study on implementation of KMC in low resource countries in Africa showed that KMC could be used for management of low-birth weight babies [7]. A multicountry randomized-controlled trial in Africa is currently evaluating the effect of immediate KMC on neonatal survival, and may provide more evidence to support the use of KMC in preventing neonatal hypothermia in low resource settings.

The statistically significant association between low Apgar scores <6 with hospital mortality reaffirms the importance of management during delivery in determining neonatal outcomes. In our setting, management of preterm infants is still lacking (no use of plastic bags, no surfactant treatment available, lack of incubators and intubation not commonly available for preterm infants). A lack of skilled healthcare workers in hospitals means that there are insufficient staff to adequately care for the mother and newborn after delivery, affecting the quality and competence of neonatal resuscitation. Reducing the incidence of hypothermia among preterm and low-birth weight infants has been evaluated in other studies [8–10]; however, while roughly 31–55% of our patients were preterm and/or had low-birth weight, over 70% of neonates were hypothermic at 5 min and on admission to the NU, indicating that there is a generally high incidence of hypothermia affecting newborns in low resource settings such as these. However, thermal care is part of basic newborn care and, if done well, can have an impact on outcomes. Therefore, reduction of neonatal mortality in countries such as Malawi can be attained through optimization of these simple measures.

The most common neonatal morbidities associated with hypothermia were RDS and birth asphyxia, and hypothermia 5 min after birth was associated with a 74% lower risk of RDS, although this association was weak after adjusting for potential confounders. Contrary to other studies, which noted the association between hypothermia and RDS in low-birth weight and preterm infants [8–10], we instead found that hypothermia 5 min after birth had an OR of 0.26 for RDS, although this association was weaker after adjusting for potential confounders [adjusted OR 0.26 (95% CI 0.07–0.99)]. Both hypothermia 5 min after birth and on admission to the NU had an OR of 0.31 and 0.32, respectively, for receipt of oxygen supplementation, although these associations were also weak after adjusting for potential confounders. These findings may reflect the constraints of staffing shortages; however, as the sample size was small, all results should be interpreted with caution. We documented, for both hypothermia at 5 min and on admission, a higher proportion of CPAP usage in both groups, although neither reached statistical significance.

The study is limited by the modest size of our sample size, which made it difficult to identify clear associations between variables, and also means that all results should be interpreted with caution. Although every effort was made to compile a complete dataset, we still had missing data, especially with labour ward temperature, and duration of time between transport from the delivery room to the NU. We did not document maternal temperature, and low staffing levels on the labour ward and NU meant there were challenges with the collection of daily observations. WHO advises rectal temperature measurements as a more accurate measure of body core temperature, when hypothermia is suspected. Given the limited equipment and staffing we were unable to do so, therefore there is a potential for misclassification of hypothermia and normothermia infants. Despite these limitations, this study provides one of the most detailed recent studies on neonatal hypothermia conducted in a low resource setting. Furthermore, we did adjust for potential confounders, and identified potential associations and interventions of significance. We identified a strong association with mortality among all neonates with hypothermia on admission to the NU; however, as all neonates who died had documented hypothermia on admission, we were unable to calculate an OR.

In conclusion, there is a high incidence of hypothermia that affects the majority of newborns at our tertiary-level hospital. Hypothermia at 5 min was associated with an OR of 13.31 for hypothermia on admission to the NU, which was itself significantly associated with mortality. Our findings reiterate the vital importance of addressing the immediate period after birth, and suggest that the use of simple interventions in the immediate period after delivery prior to arrival in the NU could improve neonatal hypothermia, and reduce associated morbidity and mortality.
